# Cocaine Cues Used in Experimental Research: A Systematic Review

**DOI:** 10.3390/brainsci15060626

**Published:** 2025-06-10

**Authors:** Eileen Brobbin, Natalie Lowry, Matteo Cella, Alex Copello, Simon Coulton, Jerome Di Pietro, Colin Drummond, Steven Glautier, Ceyda Kiyak, Thomas Phillips, Daniel Stahl, Shelley Starr, Lucia Valmaggia, Colin Williams, Paolo Deluca

**Affiliations:** 1 Addictions Department, National Addiction Centre, Institute of Psychiatry, Psychology & Neuroscience, King’s College London, London SE5 8AF, UK; natalie.m.lowry@kcl.ac.uk (N.L.); colin.drummond@kcl.ac.uk (C.D.); lucia.valmaggia@kcl.ac.uk (L.V.); paolo.deluca@kcl.ac.uk (P.D.); 2 Department of Psychology, Institute of Psychiatry, Psychology & Neuroscience, King’s College London, London SE5 8AF, UK; matteo.cella@kcl.ac.uk (M.C.); jerome.dipietro@kcl.ac.uk (J.D.P.); 3 School of Psychology, University of Birmingham, Edgbaston, Birmingham B15 2TT, UK; a.g.copello@bham.ac.uk; 4 Centre for Health Service Studies, University of Kent, Canterbury CT2 7NZ, UK; s.coulton@kent.ac.uk; 5 Department of Psychology, University of Southampton, Southampton SO17 1BJ, UK; spg@soton.ac.uk; 6 School of Psychology, University of East Anglia, Norwich NR4 7TJ, UK; c.kiyak@uea.ac.uk; 7 Centre for Addiction and Mental Health Research (CAMHR), Faculty of Health Science, University of Hull, Hull HU6 7RX, UK; thomas.phillips@hull.ac.uk; 8 Department of Biostatistics and Health Informatics, Institute of Psychiatry, Psychology & Neuroscience, King’s College London, London SE5 8AF, UK; daniel.r.stahl@kcl.ac.uk; 9 Research Team PPI Advisory Board Group, London SE5 8AF, UK

**Keywords:** cocaine-cue reactivity, cue exposure therapy, cue-induced cocaine craving, cue reactivity, cocaine, cocaine dependence, cocaine treatment

## Abstract

**Aims**: Cue exposure therapy (CET) is a promising treatment approach for cocaine substance use disorder (SUD). CET specifically targets the psychological and physiological responses elicited by drug-related cues, aiming to reduce their motivational impact. To advance understanding of CET for cocaine treatment, this systematic review aims to categorise the range of cocaine cues used in research. **Methods**: A systematic review of the existing literature with searches conducted on PubMed and Web of Science bibliographic databases with no time constraints in August 2024 (PROSPERO: CRD42024554361). Three reviewers were independently involved in the screening, review and data extraction process, in line with PRISMA guidelines. Data extracted included participant demographics, study design, data on the cocaine cue task, and examples (if provided). Each study was appraised and received a quality score. The secondary outcome was to summarise examples for each category type identified. The data are presented as a narrative synthesis. **Results**: 3600 articles were identified and screened. 235 articles were included in the analysis. Cues identified included images, paraphernalia, drug-related words, cocaine smell, auditory stimuli presented via audiotapes, video recordings, scripts, and virtual reality environments, often combining multiple modalities. Included studies recruited cocaine-dependent individuals, recreational users, polydrug users, and non-cocaine-using controls. The sample sizes of the studies ranged from a single case study to a study including 1974 participants. **Conclusions**: This review found that studies employed a wide range of cue categories, but detailed examples were often lacking, limiting replication. The number and combination of cues varied: some studies used only cocaine-related images, while others included images, videos, physical items, and audiotapes. The level of immersion and personalisation also differed considerably. All studies used cocaine-specific cues, most commonly images or representations of cocaine substance, cocaine use or drug paraphernalia, drug preparation items, or conversations of cocaine use and its effects. The overall quality of the included studies was deemed good, with all adhering to standard research norms. While this review highlights the breath of cue types used in the literature, further research should focus on enhancing cue exposure techniques by incorporating more immersive and personalised stimuli, and by providing clearer documentation of cue characteristics to support replication and clinical translation.

## 1. Background

Cocaine dependence is complex and difficult to treat, with cocaine use and related deaths in the UK, and globally, rising from previous years [1,2,3] and the global supply of cocaine at record levels [4]. There were 1118 deaths involving cocaine registered in 2023, which is 30.5% higher than in 2022 [5]. Current treatment for cocaine dependence emphasises psychological interventions such as cognitive-behavioural therapy (CBT), contingency management (CM), motivational interviewing (MI), and peer-support approaches to address behaviour change and prevent relapse [2,3,4,5,6,7]. However, treatment outcomes are often hindered by low engagement and high relapse rates, where cravings play a significant role in impeding adherence and long-term effectiveness [2,3,4,5,6]. Engagement challenges are further exacerbated by heightened reactivity to cocaine cues, triggers, and withdrawal symptoms [2].

Unlike opioid use disorder, for which effective pharmacotherapies and substitution treatments such as methadone and buprenorphine exist, there are no approved pharmacological treatments for cocaine dependence [3]. While research has explored various medications to support cocaine treatment, a systematic review concluded that medication alone was not effective for treating cocaine dependence [8]. Even though combining medication with psychosocial treatments has shown potential for reducing dropout rates, it does not significantly improve cocaine abstinence rates following treatment completion [9,10]. A critical challenge in treating cocaine dependence remains and can be addressed by reducing cravings, which is an important factor influencing relapse [2].

One promising alternative treatment approach under investigation is cue exposure therapy (CET) [6]. CET specifically targets the psychological and physiological responses elicited by drug-related cues, aiming to reduce their motivational impact. By repeatedly exposing substance-dependent individuals to these cues in a controlled environment without subsequent drug use, CET weakens the learned associations between cues and drug-related behaviours through a process known as extinction [7].

The theoretical foundation of CET is rooted in the classical conditioning model of learning, where drug-related cues become associated with drug use through repeated pairings [8]. When these cues are repeatedly presented without reinforcement (i.e., drug use), the conditioned responses, such as craving, diminish over time. In the context of SUD treatment, CET sessions involve repeated, unreinforced exposure to items, places, or actions the patient associates with drug use, in order to extinguish their previously conditioned response. By breaking the link between cue encounters and subsequent drug use, CET offers a targeted strategy for addressing drug-related triggers and reducing relapse rates [7].

Previous studies in addiction have used CET with various substance use disorders (SUD), including alcohol, nicotine, cocaine, and opiates [6,9,10,11,12,13,14,15,16,17,18]. There is some evidence for the effectiveness of CET for alcohol dependence [11,13,19,20,21] and there are emerging literature on CET for cocaine dependence [6,15,22]. CET has the potential to reduce craving and cue reactivity and, hence, reduce the risk of relapse of cocaine use [19,20], but more research is needed. There has been limited publications comparing the difference between exposure to standardised vs. personalised cues within CET substance treatment [23,24]. There is still a gap in knowledge of CET within substance use treatment of large multi-site RCTs comparing multiple cue types and investigating individual difference impact. Evidence suggests that, for successful extinction to occur, cue exposure and extinction must occur across multiple contexts to increase extinction generalisability [9], suggesting that greater variety and context provided for cues would improve treatment outcomes.

Although previous meta-analyses have shown the limited efficacy of CET for SUD treatment [9,25], this may be due to methodological limitations in CET research [26]. Many studies have presented cues individually within abstract contexts, for example, a bottle of beer on a desk in a clinic. This approach may limit generalisability to real-world drug use settings beyond the clinical environment. However, CET for SUD treatment can now take advantage of technological advances. CET research could now explore new ways to implement cues through exposure to more salient, realistic, and personally meaningful stimuli [19].

Devices such as virtual reality (VR) headsets and wearable devices enable the recording of biometric data, including heart rate variability, galvanic skin response, eye gaze, and body movements, which are biomarkers linked to substance craving. These technologies provide new ways to present multiple cues across various contexts, potentially enhancing generalisation beyond the treatment environment. These innovations should increase the generalisation of treatment effects beyond the clinical setting [26]. Notably, VR has already been successfully implemented in research to elicit and reduce alcohol cravings [26].

To advance the development of paradigms for cocaine CET, it is essential to gain a greater understanding of the types of cues that elicit craving responses, how these can be presented, and how these cues can be adapted or enhanced using emerging technologies. However, existing research on cocaine-related cues has largely overlooked the use of personalised and immersive cues in experimental studies, which are essential for improving ecological validity and replicating real-world drug-use contexts. While reviews and meta-analyses have examined CET in SUD [9,20,25], to our knowledge, no prior review has specifically focused on cocaine cues.

This paper aims to address this gap by providing a comprehensive review of cocaine cues utilised in previous cue-exposure studies. By critically evaluating these cues, we seek to inform the design and development of more effective, personalised and immersive VR-enhanced CET interventions, to reduce relapse rates and improve treatment outcomes for individuals with cocaine dependence. This review will focus on cue categories and examples within each category, including cues, situations, sense of presence and ecological validity (realism). The objectives for this review are to (1) identify the categories of cocaine craving cues used in previous research. This will include categories such as, visual cues or auditory cues, examples of cues used, how they were delivered and if they were delivered in combination with other cue types; (2) examine the range of cues utilised within each category; and (3) assess the risk of bias and quality of evidence in the included studies.

## 2. Methods

This review has been conducted according to the PRISMA 2020 guidelines (Preferred Reporting Items for Systematic Reviews and Meta-Analyses) [27]. This protocol has been registered at the International Prospective Register of Systematic Review (PROSPERO: CRD42024554361). This review was initially registered as a Rapid Review on PROSPERO; however, considering the scope and methods ultimately applied, we have revised this to a systematic review.

### 2.1. Inclusion Criteria

Studies meeting the following criteria were included: full text, original studies published in peer-reviewed journals, in English and investigating, reporting or including the use of cocaine cues. There were no restrictions on publication year or participant clinical and demographic characteristics. Cocaine-dependent individuals, recreational users, polydrug users, and non-cocaine-using controls were included and not restricted by the severity of cocaine dependence or comorbidities to provide a comprehensive understanding of cue reactivity across diverse groups. Conference abstracts, dissertations, and grey literature were not considered.

### 2.2. Information Sources

The bibliographic databases used were PubMed and Web of Science. Searches were carried out on the 2 August 2024. The search terms were adapted for use with each bibliographic database in combination with their specific filters (Table 1). The searches were supplemented by cross-checking the reference lists of key publications, related reviews, and included papers. The search terms and information sources were collaboratively developed and refined in consultation with the 15-member project team.

All papers identified in the search strategy were exported into the citation management system, Rayyan [28], and were screened at the title and/or abstract level to identify studies that potentially met the inclusion criteria (EB). From this list, the full text was retrieved and assessed independently by EB and NL, and any doubts were discussed between the first and second reviewers (EB and NL). Any disagreement between the first and second reviewers was discussed with a third reviewer (PD). Hand searching for additional papers also occurred within the already identified papers. A data extraction table was created and pilot-tested with the first five included studies and refined as necessary. EB extracted the data independently, and NL conducted entry checks for accuracy.

### 2.3. Outcomes

The primary outcome of this review was to identify categories of cocaine craving cues. The secondary outcome of this review was to identify the range of cues utilised within each category. Data extracted included study characteristics: country, publication year, source of funding, author conflicts of interest, study aims, population health status, type of cocaine, number of participants, study design, type of cues, examples, results, and any study limitations relating to cocaine cues.

### 2.4. Risk of Bias in Individual Studies

Study quality was assessed using an adapted eight-item framework [29], assessing study design, sample representativeness, measurement reliability, outcome validity, confounding control, statistical analysis, attrition and follow-up, with details provided in the Supplementary Materials. The scoring system awarded a maximum of one point for each of the eight criteria (maximum score of eight). Scores for attrition rates were adapted by allowing an incremental 0.5 points for discussion of attrition rates and an additional 0.5 points for having a follow-up rate greater than 50% [30]. The first and second reviewers (EB and NL) scored the articles independently and discussed any queries. The purpose of this quality appraisal was to ensure adherence to research standards and were not unevaluated reports of clinical innovations. The aim of this quality appraisal was not to exclude papers with lower scores but to explore all studies using cocaine cues and the range of methods and outcomes studied.

### 2.5. Data Synthesis and Analysis

Findings from the included studies are presented in a narrative synthesis. Information is included on the type of intervention (individual behaviour change, health service use), population characteristics, clinical or non-clinical population, the type of outcome and intervention content. The narrative synthesis of results on the primary outcomes, categories of cocaine craving cues, and subsequent examples are grouped by senses: Visual, tactile, auditory, gustatory and olfactory. Information on the extent of personalisation of cues and immersiveness was also identified. This review aimed to categorise and summarise examples of cocaine cues. Therefore, a meta-analysis was not appropriate within the scope of this review.

## 3. Results

**Figure 1:**
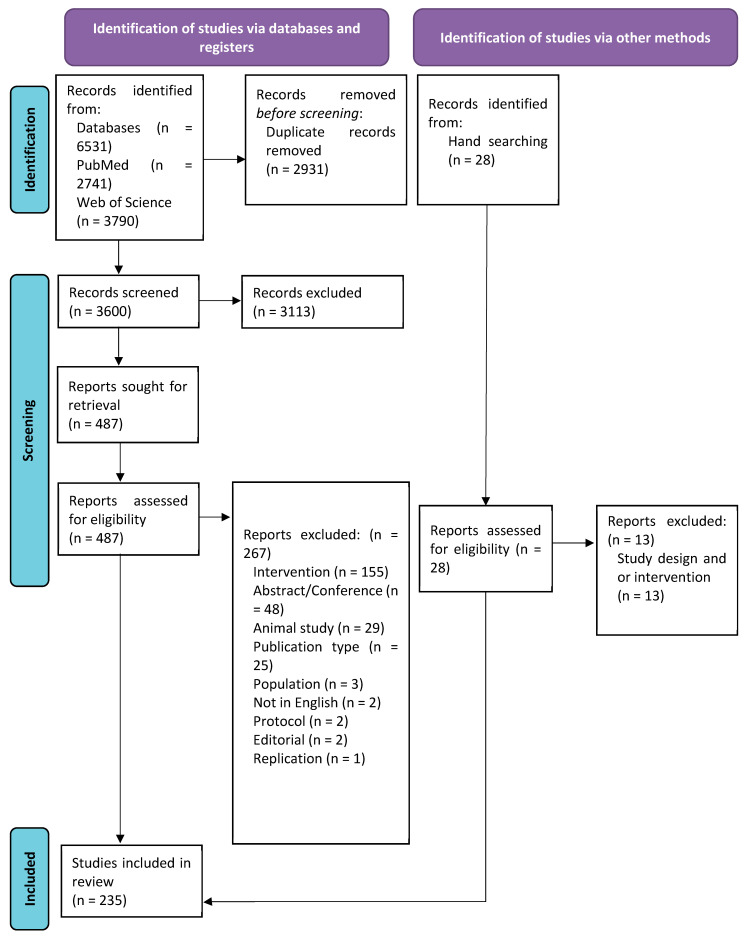
PRISMA flow diagram.

After duplications were removed, 3600 papers were screened; of these, 3113 were excluded at the title/abstract screening, and 487 full-text papers were assessed. A total of 267 papers were then excluded. There were 28 additional papers identified by hand searching; of these, 15 were included. The final sample included 235 publications (Figure 1).

### 3.1. Study Characteristics

Of the 235 included papers, most were conducted in the United States (US) (84.9%) (Table 2). The first paper was published in 1987, and over half of the studies have been published since 2010 (59.6%). Most included participants not currently in treatment and without a clinical SUD diagnosis (60.4%). The majority reported including cocaine users (85.1%), with only 30 (12.8%) specifying the type of cocaine used (powder cocaine, crack or combination with or without opioids). There were 234 quantitative studies, of which 59 were randomised controlled trials [22,31,32,33,34,35,36,37,38,39,40,41,42,43,44,45,46,47,48,49,50,51,52,53,54,55,56,57,58,59,60,61,62,63,64,65,66,67,68,69,70,71,72,73,74,75,76,77,78,79,80,81,82,83,84,85,86,87,88] and one qualitative study [89,90]. A summary of all included studies is provided in the Supplementary Materials.

A variety of cue types were used. These are reported below with example in categories of sense types: visual (n = 200 studies), auditory (n = 70 studies), tactile (n = 52 studies), olfactory (n = 5 studies) and gustatory (n = 4 studies). The most commonly used cue was a still image (83 studies, 25.7%), and the second videos (73 studies, 24.5%), the least common were VR cocaine experience (3 studies, 0.9%) and drug memory recall (1 study, 0.3%). Many studies used only one type of cue (166 studies, 70.6%) of either images only, video only, script only or words only. But other studies used combinations of two or more cue types (Table 2). Of those who report including cocaine powder and crack users or crack users only, many (20/25, 80%) reports using specific crack-related videos, images, paraphernalia, or participant-identified scenarios for guided scripts [14,39,87,88,91,92,93,94,95,96,97,98,99,100,101,102,103,104,105,106].

### 3.2. Quality Assessment

Study quality scores ranged from 4.5 to 8.0 (Mean: 6.85, Median: 7, Mode: 6.5) out of a possible 8. Only one study scored 4.5, and this was a case study [107]. Overall, the descriptive quality of the included studies was deemed good; all conformed to standard research norms and were not unevaluated reports or clinical innovations.

### 3.3. Narrative Findings

A range of sensory cues were used to trigger cocaine cravings, including visual, auditory, tactile, olfactory and gustatory cues. The following subsections provide a detailed analysis of how each sensory modality was used to trigger craving responses.

Of the 235 studies, a total of 33 (14.0%) included an example of the cue, either an image, a still image of the video, or a passage from the audio or guided script [6,35,46,50,62,70,75,106,108,109,110,111,112,113,114,115,116,117,118,119,120,121,122,123,124,125,126,127,128,129,130,131,132].

#### 3.3.1. Visual

There were 200 studies included that incorporated visual cues. These included cocaine-related videos, images, items and VR. Most studies used cocaine-related images (n = 83) [14,22,35,36,43,44,46,47,48,49,50,53,62,65,67,82,91,92,94,99,104,105,107,109,110,111,114,115,118,119,120,122,123,124,127,128,129,130,131,132,133,134,135,136,137,138,139,140,141,142,143,144,145,146,147,148,149,150,151,152,153,154,155,156,157,158,159,160,161,162,163,164,165,166,167,168,169,170,171,172,173,174,175], or videos (n = 79 studies) [31,32,33,34,37,56,57,58,61,69,70,72,73,75,76,77,79,80,81,83,85,86,87,88,91,96,97,98,100,102,103,112,125,162,176,177,178,179,180,181,182,183,184,185,186,187,188,189,190,191,192,193,194,195,196,197,198,199,200,201,202,203,204,205,206,207,208,209,210,211,212,213,214,215,216,217,218,219,220]. A total of 52 studies [34,39,40,44,51,54,57,66,68,75,76,79,80,81,83,84,85,86,87,88,96,100,101,102,103,133,134,163,176,177,178,179,180,181,182,183,184,186,187,188,189,190,191,192,193,194,221,222,223,224,225,226] had cocaine-use-related items that participants could look at, touch, or use in a drug preparation or drug use task. Cocaine-related items included crack pipes/stems, simulated powder cocaine, simulated crack rocks, lighters, banknotes, mirrors, straws, and razor blades. In studies that recruited powder or crack cocaine users, the paraphernalia often corresponded to their preferred method of administration, for example, small plastic bags and banknotes for powder cocaine users and crack pipes and lighters for crack users.

Eight studies [34,87,88,96,180,186,192,193] involved a drug preparation task in which participants were asked to repeatedly prepare lines of powder using paraphernalia (e.g., a razor blade) or to prepare a pipe or spoon with rocks. Four studies included a cocaine drug use task [101,133,193,225], while two involved placebo drug use [84,133]. These tasks required participants to view drug preparation items and cocaine or placebo cocaine; while noted here, further details on these tasks will be provided in the following sections. Additionally, three studies used VR environments and headsets to immerse participants in drug-related scenarios [6,106,108].

Examples of images include drug paraphernalia, such as crack pipes, mirrors, razor blades, straws, rolled-up money, lighters, vials, scrapers, rolling paper, plastic bags, injecting equipment, and glass stems. Images also depicted individuals using cocaine, either snorting or injecting, as well as people buying, using, or becoming intoxicated. Many images featured cocaine with general themes of drug preparation rituals, such as lines of powder next to a credit card, crack rocks next to a pipe or preparing a syringe for injection.

Videos often showed adults purchasing, using, and feeling the effects of cocaine via different administrations (snorting, smoking, injecting). Some videos featured individuals talking about their use and the rush or sensation they experienced afterwards. Studies often noted that these videos contained drug-related themes and scenes where individuals engaged in various drug-related tasks (purchasing, using, or discussing drug use). While some studies noted that videos showed drug use in various environments [81,111], very few provided detailed descriptions of the environments where drug use or purchases took place. Additionally, some videos included scenes of stress and the effects of using cocaine.

In addition to the above, there were also tasks involving viewing drug-related words, for example, when completing a Stroop task. A total of 27 studies described cues involving words or a Stroop task [63,64,78,119,130,149,168,175,227,228,229,230,231,232,233,234,235,236,237,238,239,240,241,242,243,244,245], where drug-related words were presented on a computer screen. Examples of cocaine-related words included cocaine, coke, base, high, flash, blow, pipe, and dope. Neutral words such as household items (sofa, oven, kitchen, table, book), environmental terms (north, south, east, west), and food-related words (fruit and vegetables), were often used as control conditions.

There were also some studies which did not include specific examples but referred to ‘cocaine cues’, ‘cues of images containing cocaine themes’, ‘cocaine video’ or ‘cocaine words’, and the full description of the cue was not always reported [36,49,50,58,62,64,70,82,94,107,119,128,131,139,144,154,156,166,170,174,175,184,211,219,229,230,231,233,234,237,240,241,242,243,244,245].

There was a lack of clarity on how these cues were decided or where they were sourced from. One study reported the use of cocaine scenes taken from television programmes [185]. Four studies reported using images from participants’ real drug use locations. These images were created by researchers [43,57,72] or, in one study, created by the participants themselves [53].

In studies using VR, participants were exposed to computer-generated VR environments designed to simulate cocaine-related scenarios [6,106,108]. For example, in one study [6], the participant is placed in an apartment setting when friends arrive and start talking about and using cocaine on a coffee table. The participant is offered cocaine and, at least once during the scene, uses VR hand controllers to simulate the handling and touching of virtual cocaine paraphernalia, such as a steel spoon, paper tubes, glass pipes, or syringes. Participants could also virtually prepare and self-administer cocaine within the VR environment.

#### 3.3.2. Auditory

Seventy studies incorporated auditory cues [5,11,31,35,38,39,42,48,49,50,52,54,56,57,68,71,72,77,81,82,83,85,86,94,98,102,103,107,108,117,152,166,167,169,170,173,176,178,179,180,182,183,184,185,188,190,195,205,210,229,241,242,243,244,245,246,247,248,249,250,251,252,253,254,255,256,257,258]. All 70 studies used at least one of either audiotapes, audio within videos, guided imagery scripts, or audio in VR. One of these 70 studies [166] reported using audio cues but did not specify the content or delivery of the cue.

In addition, there are also three studies which referred to script development and rehearsal of a drug experience, but it is unclear if this was then listened to [96,179,259].

A total of 13 studies used audiotapes to elicit cocaine cravings [11,34,52,53,85,116,180,186,188,190,192,194,199].

One study [199] was a 45 min long and depicted a variety of pleasurable experiences from cocaine use. Other involved individuals talking about cocaine use [12], relapse-risk situations [34,52,192], and a discussion among a group of cocaine users concerning the effects of smoking crack [34]. Similarly, five studies incorporated audiotapes depending on the route of administration (intravenous or smoking), and participants listened to patients discussing the effects of cocaine [85,186,188,190,194]. Another study had a five-minute audio recording of the participant describing craving experiences and other sounds recalled in the craving experience, such as traffic or music playing [53].

Twelve studies reported including an audio component to a video cue. Most of the videos (n = 11) included audio recordings of actors [57,86,87,88,103,125,183,197,203,212,217], and one had audio of the participant’s friend using cocaine [193]. Videos showed actors engaging in cocaine-related activities [217]; examples included an actor describing cocaine use [203], a five-minute video depicting actors purchasing and smoking crack cocaine [86,88,103], seven-minute video of actors engaging in cocaine-related activities [183], a 30 s video, of an actor purchasing, preparing, ingesting and feeling intoxicated from powder cocaine [125] and a five [57] or 25 min video, also simulating, purchasing, preparation, and smoking of crack cocaine [197]. Another example of a 3–4.5 min video that used audio from an actor spoke about perceived injustices, described the desire to get ‘high’, prepared and used fake crack cocaine, commented on being dissatisfied by the experience and teased the viewer about wanting to get ‘high’ [212]. Finally, one 60 min video had audio of the participant’s friend using cocaine [193].

Forty-one of the 45 studies which used guided imagery scripts also reported the use of listening to these to elicit cocaine cravings [38,41,42,45,51,55,60,71,74,75,80,84,89,93,95,100,113,117,121,126,135,165,178,189,246,247,248,249,250,251,252,253,254,255,256,257,258,260,261,262,263]. These were mainly delivered via a recorded audiotape of the developed guided image script; however, one study mentioned that it was read by the therapist [84], and another where the participants were instructed to think about the drug scenario through guided imagery [102]. The other three studies that used guided imagery were unclear on whether this was listened to [96,179,259]. Some described a situation where the participant had previously craved cocaine which resulted in cocaine use [38,41,55,250,251,252,253,254,255]. Examples of the guided imagery scripts include first-person and present tense guided imagery lasting approximately one minute [117]; the content of the imagery was a typical cocaine-use experience. Another imagery exercise lasted one minute and was customised depending on the route of administration and described the urge to use cocaine [71]. In some instances, the cocaine-related script was personalised [74] by including people, places, and objects [256,257]. Other forms of personalising a script included emotional and sensory cocaine-related cues and drawing upon participants’ personal drug experiences [258]. Another study [75] asked participants to create four personalised imagery scripts: past–positive, a pleasurable event from before initiation to cocaine use; past–negative, a ‘worst time’ aversive cocaine craving and use; future–positive, simulation of a ‘wished for’ event if they recovered from cocaine use disorder; future–negative, a ‘most feared’ event if cocaine use disorder worsened. Each audio lasted approximately five minutes. Other examples of a cocaine-related script were a pleasurable scenario, with associated senses [42,96,100,261] or a time when they had anticipatory excitement for cocaine [45,55,121]; one study focused on the physiologic sensations [247].

The videos, script, and audio cues included in this section include all the studies which refer to audio within these cues, either within the video or audiotape used or mention the script being read aloud.

Two studies incorporated auditory elements in VR. One study used a Meta Quest 2 VR headset, a device capable of spatial audio positioning, which was reported to enhance the sense of 360-degree immersion [6]. The other study used a VR system equipped with a VFX3D HMD, integrated stereo headphones, and a floor platform designed to enhance auditory stimuli with vibrational feedback. For instance, during a police raid scene, the floor vibrated to simulate the impact of car doors slamming or the forceful opening of doors to a crack house. The audio was set in a typical environment such as a crack house [106].

In addition to audio cues being used to elicit cocaine craving, another aspect of the audio cues in some studies was to elicit anticipation of cocaine. Anticipation-induced craving was elicited through audio scripts or direct verbal communication from the researchers about the future use of cocaine. There were five studies which reported using anticipation as a cocaine cue [89,121,226,246,260]. Four studies used anticipation in the form of an imagery script [89,121,246,260]. These scripts included memories and actions associated with anticipation of drug use [121], and describing recent situations that involved anticipatory excitement for wanting cocaine [246], and led to subsequent cocaine use [89,260]. One study used anticipation in the form of being told they were able to self-administer cocaine use (via insufflation) after the experimental procedures were completed [226].

#### 3.3.3. Tactile

A total of 52 studies incorporated tactile elements into the cocaine cue exposure [34,39,40,44,51,54,57,66,68,75,76,79,80,81,83,84,85,86,87,88,96,100,101,102,103,133,134,163,176,177,178,179,180,181,182,183,184,186,187,188,189,190,191,192,193,194,221,222,223,224,225,226]. This was achieved by providing paraphernalia and drug-related items for participants to handle, to prepare, or use, in placebo or drug-related tasks. Drug preparation tasks involved providing participants with paraphernalia and instructing them to complete a preparation task (n = 8) [34,87,88,96,180,186,192,193] or a drug use task involving cocaine or cocaine placebo (n = 4) [84,101,133,193].

Participants could touch various items, including simulated cocaine (in powder or rock form) and tools associated with drug preparation, for example, ‘dime bags’, a small bag of simulated crack or powder cocaine, crack pipe, lighter, money (typically $5 or $10 bills), mirror, straw, razor blade, rolling paper, lactose, rock candy, butane lighter, or a glass stem.

In drug preparation tasks, participants were asked to go beyond merely touching the items by performing the actions typically performed before drug use. For example, they were instructed to use a razor blade to divide the powder into lines multiple times, hold a straw, touch and smell the crystal and put them in a pipe.

#### 3.3.4. Olfactory

There were five studies included in this review that incorporated smell into the cocaine cue exposure [84,87,88,103,189]. All studies included an olfactory cocaine cue alongside other cues. Four studies involved smelling a recently used crack pipe [87,88,103,189] and the other study used a commercially available cocaine aroma and drops of this were placed in the participants’ hands to try to recreate the smell of cocaine [84].

In addition to these five studies that mention smell as a cue, other studies may have had participants also experiencing cocaine-related smells through cocaine drug use tasks, as described in the below Section 3.3.5.

#### 3.3.5. Gustatory

Four studies incorporated taste into cocaine cue exposure [114,182,219,220]. Both studies only recruited participants who used crack cocaine and asked them to smoke cocaine. Within two studies [219,220], participants were blindfolded to remove any visual cues and then given either a cocaine base formula or a placebo in a glass stem. Participants were then asked to inhale the substance with their usual smoking practices. In the third study [133], participants were also asked to smoke cocaine base or a placebo, through a glass mouthpiece silicone oil bath. The fourth study involved participants using cocaine [182] and consuming lines of powder cocaine.

## 4. Discussion

Cue exposure and its therapeutic applications can enhance treatment outcomes for individuals with addiction. This review highlights that cue exposure is widely used in cocaine use research, allowing us to identify and categorise different types of cocaine craving cues. Notably, this is the first review to systematically explore and classify these cues. Our findings have the potential to enhance the use of cues in experimental and clinical research, ultimately contributing to more effective treatment strategies.

### 4.1. Types of Cocaine Cues and Their Use in Research

A variety of cue types were used either individually or in combination, including images, videos, physical items, scripted/guided imagery, words, audiotapes, drug preparation or drug use, VR, and drug memory recall. Images were the most used cues [83 studies) [14,22,35,36,43,44,46,47,48,49,50,53,62,65,67,82,91,92,94,99,104,105,107,109,110,111,114,115,118,119,120,122,123,124,127,128,129,130,131,132,133,134,135,136,137,138,139,140,141,142,143,144,145,146,147,148,149,150,151,152,153,154,155,156,157,158,159,160,161,162,163,164,165,166,167,168,169,170,171,172,173,174,175], followed by videos (79 studies) [31,32,33,34,37,56,57,58,61,69,70,72,73,75,76,77,79,80,81,83,85,86,87,88,91,96,97,98,100,102,103,112,125,162,176,177,178,179,180,181,182,183,184,185,186,187,188,189,190,191,192,193,194,195,196,197,198,199,200,201,202,203,204,205,206,207,208,209,210,211,212,213,214,215,216,217,218,219,220]. In contrast, VR (3 studies) [6,106,108] and drug memory recall were the least used cues (one study) [51].

Most studies used only one cue (183 studies, 77.9%), while 52 studies (22.1%) incorporated a combination of two, three, or four cues. All studies included at least one of the following: cocaine images, videos, references to cocaine drug use in audiotapes, and physical drug-related items.

The level of immersion varied across studies, depending on the number of cues used and how many senses were involved during the cue exposure. Some studies used one type of cue, e.g., images of cocaine, drug paraphernalia, or people using cocaine. Others combined multiple sensory cues, such as images or videos paired with audio recordings of drug-related conversations, the smell of cocaine, or tactile interaction with paraphernalia. VR [6,106,108] was used in a few studies to create immersive drug-related environments while maintaining the safety of the research laboratory.

### 4.2. The Role of Personalisation and Ecological Validity

Most studies were conducted in laboratory settings, where participants attended sessions and were presented with standardised cues. While this approach ensures uniformity and replicability, it may not fully capture the complexity of real-world drug experiences. A few studies extended data collection to participants’ real-world settings, allowing them to document their own cues [43,53,72]. Incorporating real-world settings and participant-identified cues could capture more personalised and non-drug-specific cues. For instance, a particular song linked to drug use, someone they have used cocaine with, a specific bag used to carry drugs, a street corner associated with purchases, or the type of phone used by a dealer could serve as intensely personal cues.

There was also variation in the degree of personalisation used in different studies. Many used stock images of cocaine, paraphernalia, and drug use [122,164,168,171] while other used images from media/films portraying drug use [185]. A small number of studies attempted to personalise cues by incorporating images of local areas where participants had previously used cocaine [43,57,72], asking participants to provide personal images [53], or featuring a friend of the participant in a cocaine-use video [193]. Some studies also personalised the guided imagery scripts with the participants’ input [74,75,256,257,258].

Despite the importance of ecological validity, none of the reviewed studies explicitly mentioned incorporating the sense of presence or realism in cue design. However, five studies did include anticipation as a cocaine cue [89,121,226,246,260], using imagery scripts or telling participants they would have access to cocaine after the experiment.

### 4.3. The Importance of Multi-Sensory Cues

Cocaine craving cues engage multiple sensory modalities [264]; yet, most studies focused primarily on visual drug-related stimuli. This is supported by research showing that the brain regions associated with cravings are activated by visual drug-related cues [137,265]. However, other senses, particularly olfaction, remain underexplored in cocaine-related studies despite the evidence suggesting a strong association between smell, craving and addiction [266,267]. The under-utilisation of olfactory cues may limit the vividness and personal relevance of the cues, potentially reducing their effectiveness in eliciting realistic craving responses.

### 4.4. Cue Exposure Therapy (CET) and Future Directions

While there was a range of aims and reasons for displaying or delivering cocaine cues within these included studies, only two specifically examined its application in CET [22,66]. In both studies, participants were randomised to receive d-cycloserine (DCS) or a placebo and completed cue reactivity tasks. The findings indicated that CET reduced brain activation to drug cues within various brain regions and that extinction to drug cues occurred in both the DCS and placebo groups between cue exposures, but that DCS did not facilitate learning extinction.

CET is an area of increasing interest as a potential treatment modality for cocaine and other substance use disorders [6,9,10,11,12,13,14,15,16,17,18]. This review highlights key considerations for improving CET, particularly regarding the balance between standardised and personalised cues. While standardised cues enhance replicability, personalisation may increase ecological validity and treatment effectiveness. A practical approach to achieving both would be integrating common themes, environments, contexts and languages informed by individuals with lived experience. For instance, cues could incorporate local slang, images of commonly known drug-use locations or familiar architecture, making the cues more relatable and immersive for participants.

Patient and public involvement (PPI) was not explicitly mentioned in the reviewed studies, but its inclusion could improve cue design, ensuring relevance across socioeconomic groups. Individuals from lower SES backgrounds may not typically use drugs in settings such as pubs or nightclubs due to financial constraints, whereas higher SES groups may report substance use in these environments or at work-related events. Personalised cues tailored to these distinctions could improve the inclusivity and effectiveness of cue-based interventions [266].

Finally, this review identified three studies which used VR to deliver virtual drug cues [6,106,108]. Technological advances allow for new modes to deliver CET, for example, the use of VR headsets, controllers or the use of other technology, for example, 360 videos (immersive videos which capture a panoramic sphere in all directions). Evidence suggests that technology delivered CET may improve the efficacy of the treatment intervention; however, there also other challenges when implementing technology in treatment, including training, costs, hardware and software infrastructure, and ethical considerations [268].

### 4.5. Recommendations

Future research should prioritise the use of personalised cues to enhance ecological validity and treatment effectiveness. The level of immersion required should be carefully considered, as more immersive and individually relevant cues may elicit stronger craving responses, potentially improving the efficacy of CET. In addition, multi-sensory cue presentations, incorporating visual, auditory, tactile, olfactory, and gustatory stimuli, should be further explored to determine their potential benefits in real-world applications.

A major challenge identified in this review was the lack of detailed descriptions of cues used in many studies. While cues were often broadly categorised as drug-related, critical information, such as the specific content of scripts, the visual and auditory details of videos, and the nature of the environments depicted, was frequently missing. This lack of specificity hinders the ability of future researchers to replicate and adapt these cues for their own studies, ultimately limiting progress in the field. To address this issue, future research should provide more comprehensive descriptions of the cues used, including their sensory characteristics and contextual details, to improve reproducibility, comparability, and the development of more effective CET protocols.

There is also a need for greater standardisation in cue exposure research. Establishing international consensus on the selection and categorisation of cues, particularly in relation to their level of immersiveness and personalisation, would help to improve methodological consistency across studies. Standardised categories could be developed to distinguish between cues used in experimental cue exposure research and those employed in CET interventions. Using open-source methodologies or equivalent initiatives could provide a transparent framework for researchers to share details of their cue exposure paradigms. Such an approach would facilitate collaboration, enhance methodological rigour, and accelerate advancements in CET research.

### 4.6. Limitations

Although we reviewed a large body of evidence, publication bias may still be present, as studies with positive findings are more likely to be published in peer-reviewed journals or English-language journals. Relevant studies published in non-English literature may have been missed. In addition, this review is limited by the scope of this work. The results presented describe the characteristics of studies involving cue exposure and measurements of craving. However, it does not provide a critical appraisal or address specific questions about the included studies, effects of cue exposure, effectiveness of cocaine cues, or whether features or cue modalities are more impactful than others.

## 5. Conclusions

This review identified a range of sensory cues used in studies to elicit cocaine craving, encompassing all the senses: visual, tactile, auditory, olfactory, and gustatory. The number and type of cues varied across the studies, but all included at least one cocaine-specific cue. Common examples included images of substances representing cocaine, paraphernalia, cocaine preparation items, talk of cocaine use, and images or videos of others using cocaine.

Due to the laboratory settings for most of these studies and the use of standardised cues for all participants, personalisation was limited, with many cues sourced from stock images. As some research suggests that CET might be effective for substance use disorders [6,9,10,11,12,13,14,15,16,17,18] and specifically for cocaine [6,15,22], further research should investigate some critical aspects that may influence its effectiveness, including the role of multiple sensory cues, personalisation, and ecological validity.

## Figures and Tables

**Table 1:** Search terms.

PubMed	Web of Science (#1)	Web of Science (#2)
TITLE/ABSTRACT: (“Cocaine” OR “crack” OR “coke”)	TITLE: (“Cocaine” OR “crack” OR “coke”)	ABSTRACT: (“Cocaine” OR “crack” OR “coke”)
AND	AND	AND
TITLE/ABSTRACT: (“cue reactivity” OR “cue” OR “craving” OR “trigger” OR “urge” OR “lapse” OR “priming”)	TITLE: (“cue reactivity” OR “cue” OR “craving” OR “trigger” OR “urge” OR “lapse” OR “priming”)	ABSTRACT: (“cue reactivity” OR “cue” OR “craving” OR “trigger” OR “urge” OR “lapse” OR “priming”)
NOT	NOT	NOT
TITLE/ABSTRACT: (“Animal”)	TITLE: (“Animal”)	ABSTRACT: (“Animal”)

**Table 2:** Study characteristics.

Study Characteristics	N = 235 (%)
**COUNTRY**	
United States	199 (84.7%)
Canada	8 (3.4%)
Netherlands	8 (3.4%)
Brazil	5 (2.1%)
United Kingdom	4 (1.7%)
France	3 (1.3%)
Italy	3 (1.3%)
Spain	3 (1.3%)
Switzerland	2 (0.9%)
**PUBLICATION YEAR**	
2016–2025	73 (31.1%)
2006–2015	98 (41.7%)
1996–2005	50 (21.3%)
1987–1995	14 (6.0%)
**PARTICIPANT GROUP**	
Non-clinical/not in treatment	138 (58.7%)
Clinical/in treatment	83 (35.3%)
Clinical and non-clinical	9 (3.8%)
Incarcerated offenders	4 (1.7%)
Not reported	1 (0.4%)
**COCAINE TYPE**	
Cocaine	200 (85.1%)
Crack	25 (10.6%)
Cocaine and opioid user	6 (2.6%)
Cocaine and crack	4 (1.7%)
**STUDY AIM**	
Cocaine task for brain imaging	85 (36.2%)
To assess medication as part of cocaine SUD treatment	46 (19.6%)
To assess cue-induced craving or drug use	44 (18.7%)
Cocaine cue task for attentional bias	30 (12.8%)
To assess treatment for cocaine SUD (with the use of cues)	13 (5.5%)
Cocaine cues for choice task	4 (1.7%)
Discussion of drug cues	3 (1.3%)
Gene testing study	3 (1.3%)
To test cocaine cues in VR	2 (0.9%)
To assess memory recall	1 (0.4%)
To assess inhibitory control	1 (0.4%)
To assess immune system cytokines	1 (0.4%)
To assess verbal fluency	1 (0.4%)
To assess cocaine induced psychosis	1 (0.4%)
**CUE TYPES**	N = 323 * (%)
Image	83 (25.7%)
Video	79 (24.5%)
Items (paraphernalia)	52 (16.1%)
Script/guided imagery	45 (14.0%)
Words (e.g., Stroop task)	27 (8.4%)
Audiotape	14 (4.3%)
Drug preparation task	8 (2.5%)
Drug use or placebo drug use	6 (1.9%)
Olfactory	5 (1.6%)
VR	3 (0.9%)
Drug memory recall	1 (0.3%)
**CUE COMBINATIONS**	N = 235 (%)
Image only	69 (29.4%)
Video only	41 (17.4%)
Script only	34 (14.5%)
Words only	22 (9.4%)
Video and items	12 (5.1%)
Items only	11 (4.7%)
Video, audio and items	6 (2.6%)
Video, script and items	6 (2.6%)
Image and words	5 (2.1%)
Image and items	3 (1.3%)
Video, audio and drug prep	3 (1.3%)
VR only	3 (1.3%)
Audio only	2 (0.9%)
Image and script	2 (0.9%)
Video and image	2 (0.9%)
Video, items, drug prep task and olfactory	2 (0.9%)
Drug use only	1 (0.4%)
Image and audio	1 (0.4%)
Image, placebo drug use and drug use	1 (0.4%)
Items and drug memory recall	1 (0.4%)
Items and drug use	1 (0.4%)
Script, items, olfactory and placebo drug use	1 (0.4%)
Video and audio	1 (0.4%)
Video, audio, items and drug prep task	1 (0.4%)
Video, item, drug prep task and drug use	1 (0.4%)
Video, items and olfactory	1 (0.4%)
Video, script, items and drug prep task	1 (0.4%)
Video, script, items and olfactory	1 (0.4%)

* Some studies used more than one cue. SUD = substance use disorder. VR = virtual reality.

## Supplementary Materials

The following supporting information can be downloaded at: https://www.mdpi.com/article/10.3390/brainsci15060626/s1, File S1: Included studies.

## Abbreviations

CBT	Cognitive behavioural therapy
CET	Cue exposure therapy
DCS	d-cycloserine
MI	Motivational interviewing
MET	Motivational enhancement therapy
OST	Opioid substitution treatment
OUD	Opioid use disorder
PPI	Patient and public involvement
PRISMA-P	Preferred Reporting Items for Systematic Reviews and Meta-Analyses
PROSPERO	Prospective Register of Systematic Review
SUD	Substance use disorder
US	United States
UK	United Kingdom
VR	Virtual reality
