# Supplementary material for: Cocaine Cues Used in Experimental Research: A Systematic Review
Source: Brain Sci. 2025 Jun 10;15(6):626. doi: 10.3390/brainsci15060626 (PMC12191070; doi:10.3390/brainsci15060626)
Supplement: Supplementary file 1 [file brainsci-15-00626-s001.zip › brainsci-3634940-supplementary.pdf]

Supplementary Materials.

Included studies (N=235).

Author and year	Aim	Study design	Participants	Type of cocaine	Cue	Population	Quality appraisal *	Sense
**Alcorn et al. 2017**	Cocaine task for attentional bias and cocaine task for eye tracking	Exploratory study	N = 20	Cocaine	Images	Nonclinical	7.5	Visual
**Alcorn et al. 2020**	Cocaine task for eye-tracking and to assess medication as part of cocaine treatment	Within-subject, outpatient, acute dosing study	N = 12	Cocaine	Images	Clinical	6.5	Visual
**Alim et al. 1995**	To assess medication as part of cocaine treatment	2-week double-blind, double-dummy, parallel-group comparison of four dosage levels	N = 50	Crack	Images	Nonclinical	7.5	Visual
**Alves et al. 2012**	Assessing treatment for cocaine dependence (with the use of cues)	Exploratory study	N = 30	Cocaine and crack	Images and videos	Nonclinical	6.5	Visual
**Anastasio et al. 2014**	Gene testing	Translational study	N = 114	Cocaine	Drug attentional bias task (Stroop test)	Nonclinical	7	Visual
**Antons et al. 2023**	Cocaine task for brain activity	Exploratory, neuroimaging study	N = 52 (n=28 CD, n=24 GD)	Cocaine	Videos	Nonclinical	7	Visual
**Araujo et al. 2015**	Assess cue-induced craving	Quasi-experimental study	N = 210 (n=70 crack, n=70 tobacco, n=70 marijuana)	Crack	Images	Clinical	7	Visual
**Avants et al. 1995**	Assess cue-induced craving	Exploratory study	N = 69	Cocaine	Paraphernalia and videos	Clinical	6.5	Visual<br>Tactile
**Back et al. 2010**	Assess cue-induced craving	Exploratory study	N = 53	Cocaine	Paraphernalia and videos	Nonclinical	7	Visual<br>Tactile

**Bardeen et al. 2014**	Cocaine task for attentional bias	Exploratory study	N = 58 (n=22 with BPD, n=36 without BPD)	Cocaine	Images and script	Clinical	6.5	Visual Auditory
**Bauer & Kranzler 1994**	Cocaine task for brain activity	Exploratory, neuroimaging study	N = 34 (n=18 CD, n=16 ND)	Cocaine	Videos	Nonclinical	6.5	Visual
**Becker et al. 2020**	To assess medication as part of cocaine treatment	Randomised, double-blind study	N = 33	Cocaine	Script	Nonclinical	6.5	Auditory
**Bell et al. 2014**	Cocaine task for brain activity	Exploratory, neuroimaging study	N = 20	Cocaine	Images	Clinical and nonclinical		Visual
**Berger et al. 1987**	To assess medication as part of cocaine treatment	Randomised, single-dose, crossover, placebo-controlled design	N = 20	Cocaine	Paraphernalia and videos	Clinical		Visual Auditory Tactile
**Bergquist et al. 2010**	Discussion of drug cues	Exploratory study	N = 56	Cocaine	Script	Nonclinical	7	Auditory
**Bonson et al. 2002**	Cocaine task for brain activity	Exploratory, neuroimaging study	N = 11	Cocaine	Paraphernalia, script and videos	Nonclinical	6.5	Visual Auditory Tactile
**Bordnick et al. 2004**	Assessing treatment for cocaine dependence (with the use of cues)	Between-groups study	N = 70	Cocaine	Drug use (placebo powder and crack), paraphernalia, script and smell	Clinical	7.5	Visual Auditory Tactile Olfactory
**Callans et al. 2024**	To assess medication as part of cocaine treatment and cocaine task for brain activity	Randomised, double-masked, placebo-controlled clinical trial	N = 9	Cocaine	Videos	Nonclinical	7.5	Visual

**Campbell et al. 2019**	To assess medication as part of cocaine treatment	Double-blind study	N = 23	Cocaine	Audiotape, paraphernalia and videos	Nonclinical	6.5	Visual Auditory Tactile
**Carpenter et al. 2012**	Cocaine task for attentional bias	Exploratory study	N = 25	Cocaine	Drug attentional bias task (Stroop test)	Clinical	6.5	Visual
**Ceceli et al. 2023**	Cocaine task for brain activity	Comparative neuroimaging study	N = 52 (n=26 CUD, n=26 control)	Cocaine	Drug attentional bias task (Stop-Go)	Nonclinical	6.5	Visual
**Chaplin et al. 2010**	Assess cue-induced craving	Comparative exploratory	N = 165 (n=52 AD, n=45 CD, n=68 HC)	Crack	Script	Nonclinical	7	Auditory
**Childress et al. 1999**	Cocaine task for brain activity	Comparative neuroimaging study	N = 20 (n=14 past cocaine users, n=6 controls)	Cocaine	Videos	Clinical	6	Visual Auditory
**Childress et al. 2008**	Cocaine task for brain activity	Exploratory, neuroimaging study	N = 22	Cocaine	Images	Clinical	6	Visual
**Coffey et al. 2002**	Assess cue-induced craving	Comparative, exploratory study	N = 75 (n= =30 CD + PTSD, n=45 AD + PTSD)	Crack	Images	Clinical	6.5	Visual
**Conti et al. 2014**	Cocaine task for brain activity	Randomised, sham-controlled neuro-navigated rTMS study design	N = 13	Crack	Images	Clinical	7.5	Visual
**Cope et al. 2014**	Cocaine task for brain activity	Exploratory, neuroimaging study	N = 137 (incarcerated)	Cocaine	Images	Clinical (criminal)	6.5	Visual
**Copersino et al. 2004**	Cocaine task for attentional bias	Comparative, exploratory study	N = 20	Cocaine	Drug attentional bias task (Stroop test)	Clinical	7	Visual

**Cox et al. 2017**	Cocaine task for brain activity	Exploratory, neuroimaging study	N = 9	Cocaine	Drug preparation task, drug use and videos	Nonclinical	6	Visual<br>Auditory<br>Tactile
**D'Amour-Horvat et al. 2022**	Cocaine task for brain activity	Comparative, neuroimaging study	N = 24 (n=19 CU, n=12 H)	Cocaine	Videos	Nonclinical	6.5	Visual<br>Auditory
**Dackis et al. 1987**	To assess medication as part of cocaine treatment	Randomised, double-blind, placebo-controlled study	N = 13	Cocaine	Paraphernalia	Nonclinical	7	Visual<br>Tactile
**Dakwar et al. 2014**	Assess cue-induced craving	Crossover, double-blind trial	N = 8	Crack	Paraphernalia	Nonclinical	7.5	Visual<br>Tactile
**Denomme & Shane 2020**	Cocaine task for brain activity	Comparative, neuroimaging study	N = 101 (n=57 ND, n=24 NW, n=20 W)	Cocaine	Videos	Nonclinical (criminal)	7	Visual
**DeSantis et al. 2009**	Assess cue impact on drug use	Exploratory study	N = 45	Cocaine	Paraphernalia and videos	Nonclinical	7	Visual<br>Tactile
**Devito et al. 2018**	Assessing treatment for cocaine dependence (with the use of cues)	Randomised controlled trial	N = 140	Cocaine (on methadone treatment)	Words	Clinical	7.5	Visual
**Dias et al. 2015**	Cocaine task for attentional bias and Cocaine task for eye tracking	Comparative, exploratory study	N = 87 (n=46 CU, n=41 H)	Cocaine	Images	Nonclinical	7	Visual
**Díaz-Batanero et al. 2018**	Cocaine task for attentional bias	Longitudinal study	N = 71	Cocaine	Images	Clinical	6.5	Visual
**DiGirolamo et al. 2015**	Cocaine task for eye-tracking	Exploratory study	N = 30	Cocaine	Paraphernalia	Clinical	7	Visual<br>Tactile
**DiGirolamo et al. 2017**	Cocaine task for eye-tracking	Exploratory study	N = 24	Crack	Image	Clinical	6.5	Visual
**Dudish-Poulson et al. 1997**	Assess cue impact on drug use	Placebo-controlled, cross-over study	N = 11	Crack	Drug use (placebo) and images	Nonclinical	6.5	Visual<br>Tactile<br>Gustatory

**Duncan et al. 2007**	Cocaine task for brain activity	Exploratory, neuroimaging study	N = 10	Crack	Script	Nonclinical	6.5	Auditory
**Dunning et al. 2011**	Cocaine task for brain activity	Comparative, exploratory study	N = 84 participants (n=55 CUD abstinent and current, n=29 healthy)	Cocaine	Images	Nonclinical	7	Visual
**Ehrman et al. 1992**	Assess cue-induced craving	Exploratory study	N = 15	Cocaine	Audiotape, drug preparation task and videos	Nonclinical	6.5	Visual Auditory Tactile
**Ehrman et al. 1996**	To assess medication as part of cocaine treatment	Double-blind placebo-controlled study	N = 45	Cocaine	Audiotape, paraphernalia and videos	Clinical	8	Visual Auditory Tactile
**Ehrman et al. 1998**	Assess cue impact on drug use	Exploratory study	N = 69	Cocaine	Audiotape, paraphernalia and videos	Clinical	6.5	Visual Auditory Tactile
**Elton et al. 2015**	Cocaine task for brain activity	Comparative, neuroimaging study	N = 38	Cocaine	Scripts	Nonclinical	6.5	Auditory
**Engeli et al. 2023**	Cocaine task for brain activity	Randomised, double-blind, placebo-controlled, counterbalanced crossover study	N = 41	Cocaine	Videos	Nonclinical	8	Visual
**Fernández-Calderón et al. 2021**	Cocaine task for attentional bias	Exploratory study	N = 70	Cocaine	Images	Clinical	6.5	Visual
**Foltin & Fischman 1997**	Assess cue-induced craving	Exploratory study	N = 9	Cocaine (smoking)	Paraphernalia	Nonclinical	6.5	Visual Tactile

**Fotros et al. 2013**	Cocaine task for brain activity	Exploratory, neuroimaging study	N = 12	Cocaine and crack	Drug preparation task, paraphernalia, script and videos	Nonclinical	7	Visual Tactile
**Fox et al. 2005**	Assess cue-induced craving	Mixed design	N = 54	Cocaine	Script	Nonclinical	7	Auditory
**Fox et al. 2006**	Assess cue-induced craving	Mixed design	N = 50	Cocaine	Script	Nonclinical	7	Auditory
**Fox et al. 2008**	Assess cue-induced craving	Comparative, exploratory study	N = 80 (n=40 CU, n=40 controls)	Cocaine	Script	Nonclinical	7	Auditory
**Fox et al. 2009**	Assess cue-induced craving	Mixed design	N = 84 (n=42 CU, n=42 H)	Cocaine	Script	Nonclinical	7	Auditory
**Fox et al. 2012**	To assess immune system cytokines	Mixed design	N = 55 (n=28 CD, n=27 alcohol social)	Cocaine	Script	Nonclinical	6.5	Auditory
**Fox et al. 2012**	To assess medication as part of cocaine treatment	Randomised, double-blind, placebo-controlled laboratory study	N = 29	Cocaine	Script	Nonclinical	7.5	Auditory
**Fox et al. 2013**	To assess medication as part of cocaine treatment	Randomised, double-blind, placebo-controlled laboratory study	N = 42	Cocaine	Script	Nonclinical	7.5	Auditory
**Fox et al. 2013**	Assess cue-induced craving	Mixed repeated measures design	N = 85 (n=59 CD and AD, n=26 socially drinking HC)	Cocaine	Script	Nonclinical	7	Auditory
**Fox et al. 2014**	To assess medication as part of cocaine treatment	Randomised, double-blind, placebo-controlled laboratory study	N = 40	Cocaine	Script	Nonclinical	7.5	Auditory

**Franken et al. 2000**	Cocaine task for attentional bias	Exploratory, pilot study	N = 16	Cocaine	Words	Clinical	5.5	Visual
**Franken et al. 2004**	Cocaine task for brain activity	Exploratory, neuroimaging study	N = 21	Cocaine	Images	Clinical	6.5	Visual
**Franken et al. 2008**	Cocaine task for brain activity	Comparative, neuroimaging study	N = 39 (n=23 CU, n=16 controls)	Cocaine	Images	Clinical	6.5	Visual
**Garavan et al. 2000**	Assess cue-induced craving	Comparative, neuroimaging study	N = 42 (n=24 CD, n=18 H)	Crack	Videos	Nonclinical	7	Visual
**Gardini et al. 2009**	Cocaine task for attentional bias	Comparative, exploratory study	N = 94 (n=24 CD, n=45 Heroin, n=25 H)	Cocaine	Drug attentional bias task (Stroop test)	Clinical	5.5	Visual
**Gervilla et al. 2022**	To test cocaine cues in virtual reality and assessing treatment for cocaine dependence (with the use of cues)	Exploratory study	N = 10	Cocaine	VR drug environments	Nonclinical	6.5	Visual
**Giasson-Gariépy et al. 2017**	Assess cue-induced craving	Comparative, exploratory pilot study	N = 28 (n=12 CU, n=16 CU and cannabis user)	Cocaine	Videos	Nonclinical	7	Visual
**Goldstein et al. 2007**	Cocaine task for attentional bias and cocaine task for brain activity	Comparative, neuroimaging study	N = 14	Cocaine	Drug attentional bias task (Stroop test)	Nonclinical	6	Visual
**Goldstein et al. 2007**	To assess verbal fluency	Comparative, exploratory study	N = 42	Cocaine	Words	Nonclinical	6.5	Visual
**Goldstein et al. 2009**	Cocaine task for brain activity	Comparative, neuroimaging study	N = 34 (n=17 CD, n=17 H)	Cocaine	Words	Nonclinical	6.5	Visual
**Goldstein et al. 2009**	Cocaine task for brain activity	Comparative, neuroimaging study	N = 30 (n=15 CU, n=15 H)	Cocaine	Drug attentional bias task (Stroop test)	Nonclinical	6.5	Visual
**Goldstein et al. 2010**	Cocaine task for brain activity	Placebo-controlled, counterbalanced,	N = 13	Cocaine	Words	Nonclinical	6.5	Visual

		neuroimaging study						
**Gómez-Bujedo et al. 2019**	Cocaine task for attentional bias	Comparative, exploratory study	N = 186	Cocaine	Images and words	Clinical	7	Visual
**Goudriaan et al. 2013**	Cocaine task for brain activity	Randomised placebo-controlled cross-over study using pharmacologic al fMRI	N = 13	Cocaine	Images	Clinical	7.5	Visual
**Grant et al. 1996**	Cocaine task for brain activity	Comaprative, exploratory study	N = 18 (n=13 CU, n=5 H)	Cocaine	Handle paraphernalia	Nonclinical	6.5	Visual Auditory Tactile
**Haney et al. 2021**	To assess medication as part of cocaine treatment	Exploratory study	N = 16	Cocaine (smoking)	Drug use and cues (unspecified)	Nonclinical	6.5	Visual Tactile Gustator y
**Haeny et al. 2024**	Discussion of drug cues	Comparative, exploratory study	N = 36 (n=12 AD, n=12 CD, n=12 H)	Cocaine	Script	Nonclinical	7	Auditory
**Hanlon et al. 2018**	Cocaine task for brain activity	Comparative, neuroimaging study	N = 156 (n=55 CU, n=53 alcohol, n=48 nicotine)	Cocaine	Images	Nonclinical	7	Visual
**Harris et al. 2004**	To assess medication as part of cocaine treatment	Controlled clinical trial	N = 22	Cocaine	Paraphernalia and videos	Nonclinical	7	Visual Auditory Tactile
**Hersh et al. 1995**	To assess medication as part of cocaine treatment	Randomised, double-blind, placebo-controlled trial	N = 50 (n=33 CD, n=7 H)	Cocaine	Videos	Nonclinical	7.5	Visual

**Hester et al. 2006**	Cocaine task for attentional bias	Comparative, exploratory study	N = 46 (n=23 cocaine users, n=HC)	Cocaine	Drug attentional bias task (Stroop test) and image	Nonclinical	6.5	Visual
**Hester & Garavan 2009**	Cocaine task for brain activity	Neuroimaging study	N = 16	Cocaine	Images	Nonclinical	6.5	Visual
**Hochheimer et al. 2023**	Assess cue-induced craving	Exploratory study	N = 1974	Cocaine	Images	Clinical	7	Visual
**Horrell et al. 2010**	Cocaine task for brain activity	Neuroimaging study	N = 10	Cocaine	Images	Clinical	6.5	Visual
**Jobes et al. 2011**	To assess medication as part of cocaine treatment	Randomised, double-blind study	N = 59	Cocaine	Script	Clinical	8	Auditory
**Jobes et al. 2015**	To assess medication as part of cocaine treatment	Randomised, double-blind study	N = 33	Heroin dependent (using cocaine)	Script	Clinical	7.5	Auditory
**Johnson et al. 1998**	Assess cue-induced craving	Exploratory study	N = 30	Cocaine	Audiotape, paraphernalia and videos	Nonclinical	7	Visual Auditory Tactile
**Johnson et al. 2020**	To assess medication as part of cocaine treatment	Randomised, double-blind, placebo-controlled trial	N = 39 (n=21 DCS, n=18 placebo)	Cocaine	Images	Nonclinical	7.5	Visual
**Joseph et al. 2019**	Cocaine task for brain activity	Randomised, counterbalanced crossover study	N = 67	Cocaine	Images	Nonclinical	7.5	Visual
**Kaag et al. 2018**	Cocaine task for brain activity	Comparative, exploratory study	N = 117 (n=59 CU, n=58 controls)	Cocaine	Images	Nonclinical	7	Visual
**Kearney-Ramos et al. 2018**	Cocaine task for brain activity	Two single-blinded, within-subject, active sham-controlled experiments	N = 25 (in the cocaine study)	Cocaine	Images	Nonclinical	6.5	Visual

**Kearney-Ramos et al. 2019**	Cocaine task for brain activity	Single-blind, active sham-controlled crossover study	N = 25	Cocaine	Images	Nonclinical	7	Visual
**Kennedy et al. 2014**	Cocaine task for attentional bias	Exploratory study	N = 35	Cocaine	Drug attentional bias task (Stroop test)	Clinical	7	Visual
**Kexel et al. 2022**	Assess cue-induced craving	Comparative, exploratory study	N = 123 (n=69 CU, n=54 H)	Cocaine	Videos	Nonclinical	7	Visual
**Kilgus & Pumariega 1994**	Assess cue-induced craving	Exploratory study	N = 13	Crack	Videos	Clinical	7.5	Visual
**Killeen & Brady 2000**	Assess cue-induced craving	Exploratory study	N = 30	Cocaine and crack	Videos	Clinical	6	Visual
**Kilts et al. 2001**	Cocaine task for brain activity	Comparative, neuroimaging study	N = 8	Cocaine	Script	Clinical	6.5	Auditory
**Kilts et al. 2004**	Cocaine task for brain activity	Repeated measures	N = 8	Cocaine	Script	Clinical and nonclinical	7	Auditory
**Kober et al. 2016**	Cocaine task for brain activity	Comparative, neuroimaging study	N = 103 (n=30 CD, n=28 PG, n=45 control)	Cocaine	Videos	Nonclinical	7	Visual Auditory
**Konova et al. 2019**	Cocaine task for brain activity	Comparative, neuroimaging study	N = 33 (n=18 chronic CU, n=15 matched controls)	Cocaine	Images	Nonclinical	7	Visual
**Kosten et al. 2006**	Cocaine task for brain activity	12-week, randomised, placebo-controlled, double-blind clinical trial	N = 33	Cocaine	Videos	Nonclinical	7.5	Visual
**Kranzler & Bauer 1992**	To assess medication as part of cocaine treatment	Randomised, double-blind,	N = 20	Cocaine	Videos	Clinical	7.5	Visual

		placebo-controlled study						
**Lam et al. 2013**	Cocaine task for brain activity	Exploratory study	N = 19	Cocaine	Videos	Nonclinical	6.5	Visual
**Land et al. 2020**	To assess medication as part of cocaine treatment	Exploratory study	N = 101 (n=53 CU, n=48 HC)	Cocaine	Drug attentional bias task (Stroop test)	Nonclinical	7	Visual
**LaRowe et al. 2007**	Assess cue-induced craving and to assess medication as part of cocaine treatment	Double-blind placebo-controlled trial	N = 15	Cocaine	Images	Nonclinical	6.5	Visual
**Lehoux et al. 2024**	To test cocaine cues in virtual reality	Feasibility study	N = 11	Cocaine	VR drug environments	Nonclinical	6.5	Visual Auditory
**Leyton et al. 2005**	To assess medication as part of cocaine treatment	Randomised, double-blind, controlled trial	N = 8	Cocaine	Paraphernalia	Nonclinical	6.5	Visual Tactile
**Li et al. 2005**	Cocaine task for brain activity	Experimental, neuroimaging study	N = 11	Cocaine	Script	Nonclinical	6.5	Auditory
**Li et al. 2005**	Cocaine task for brain activity	Experimental, neuroimaging study	N=27	Cocaine	Script	Nonclinical	6.5	Auditory
**Lui et al. 1998**	Cocaine task for brain activity	Experimental, neuroimaging study	N = 17	Cocaine	Paraphernalia and videos	Nonclinical	6	Visual Tactile
**Liu et al. 2011**	Cocaine task for attentional bias	Experimental study	N = 69 (n=37 CU, n=32 HC)	Cocaine	Drug attentional bias task (Stroop test)	Nonclinical	7	Visual
**Liu et al. 2013**	To assess medication as part of cocaine treatment	Double-blind placebo-controlled trial	N = 23	Cocaine	Drug attentional bias task (Stroop test)	Nonclinical	7.5	Visual
**Lowry et al. 2021**	Assess cue-induced craving	Mixed methods RCT	N = 31	Cocaine	Audiotape, paraphernalia and videos	Clinical	7.5	Visual Auditory Tactile

**Ma et al. 2018**	Cocaine task for brain activity	Within-group study	N = 15	Cocaine	Drug attentional bias task (Stroop test)	Clinical	6.5	Visual
**Ma et al. 2022**	Cocaine task for brain activity	Within-group study	N = 28	Cocaine	Drug attentional bias task (Stroop test)	Nonclinical	8	Visual
**Maas et al. 1998**	Cocaine task for brain activity	Comparative, neuroimaging study	N = 12 (n=6 crack, n=6 H)	Crack	Videos	Nonclinical	6.5	Visual
**Mahoney et al. 2020**	Assessing treatment for cocaine dependence (with the use of cues)	Case study	N = 1 (case study)	Cocaine (and heroin)	Images	Clinical	4.5	Visual
**Mahoney et al. 2023**	Cocaine task for brain activity	Open-label clinical trial	N = 4	Opioid	Images and videos	Clinical	6.5	Visual
**Margolin et al. 1994**	Assessing treatment for cocaine dependence (with the use of cues)	Experimental study	N = 19	Cocaine	Videos	Clinical	6.5	Vis[illegible]
**Marks et al. 2014**	Cocaine task for attentional bias	Between-subject, repeated measures study	N = 30 (m=15 CU, n=15 H)	Cocaine	Drug attentional bias task (Stroop test) and images	Nonclinical	7	Visual
**Marks et al. 2014**	Cocaine task for attentional bias	Within-subject, repeated measures study	N = 36	Cocaine	Images	Nonclinical	6.5	Visual
**Marks et al. 2015**	Cocaine task for eye-tracking	Experimental study	N = 20	Cocaine	Images	Nonclinical	7	Visual
**Marks et al. 2015**	Cocaine task for attentional bias	Double-blind, placebo-controlled within-subject study	N = 40 (n=20 CU, n=20 CU + alcohol)	Cocaine	Images	Nonclinical	6.5	Visual
**Marks et al. 2016**	Cocaine task for attentional bias and cocaine task for eye tracking	Experimental study	N = 40 (n=20 CU + cigarette, n=20 cigarette)	Cocaine (smoking)	Images	Nonclinical	6.5	Visual

Commented [EB1]: Links to Avants 1993

**Marsden et al. 2018**	Assessing treatment for cocaine dependence (with the use of cues)	External pilot randomised controlled trial	N = 30	Cocaine	Audiotape and images	Clinical	7.5	Visual Auditory
**Martinotti et al. 2022**	Cocaine task for brain activity and assessing treatment for cocaine dependence (with the use of cues)	Randomised, double-blind, sham-controlled trial	N = 90 (n=42 active rTMS, n=38 sham)	Cocaine	Videos	Clinical	7	Visual
**Mayer et al. 2016**	Cocaine task for attentional bias	Randomised controlled trial	N = 37	Cocaine	Images	Nonclinical	7.5	Visual
**Mayer et al. 2020**	Cocaine task for attentional bias and to assess medication as part of cocaine treatment	Randomised controlled trial	N = 37	Cocaine	Images	Nonclinical	7.5	Visual
**Meneses-Gaya et al. 2021**	Assess cue-induced craving	Randomised, double-blind, placebo-controlled trial	N = 31	Cocaine	Videos	Clinical	8	Visual
**Milella et al. 2016**	Cocaine task for brain activity	Experimental neuroimaging study	N = 12	Cocaine	Paraphernalia, script and videos	Nonclinical	7	Visual Tactile
**Milivojevic et al. 2016**	To assess medication as part of cocaine treatment	Randomised, double-blind, placebo-controlled trial	N = 46	Cocaine	Script	Nonclinical	8	Auditory
**Milivojevic et al. 2017**	To assess medication as part of cocaine treatment	Randomised, double-blind, placebo-controlled trial	N = 40	Cocaine	Script	Clinical	7.5	Auditory
**Milivojevic et al. 2022**	To assess medication as part of cocaine treatment	Randomised, double-blind, placebo-controlled trial	N = 30	Cocaine	Script	Nonclinical	8	Auditory
**Modesto-Lowe et al. 1997**	To assess medication as part of cocaine treatment	Randomised, placebo-controlled trial	N = 26	Cocaine	Videos	Nonclinical	7	Visual

**Moeller et al. 2009**	Cocaine cues for choice task	Comparative, exploratory study	N = 40 (n=CD, n=20 H)	Cocaine	Drug attentional bias task (picture choice)	Nonclinical	7	Visual
**Moeller et al. 2010**	Cocaine cues for choice task	Comparative, exploratory study	N = 65 (n=42 CD, n=23 H)	Cocaine	Drug attentional bias task (picture choice)	Nonclinical	7	Visual
**Moeller et al. 2012**	Cocaine cues for choice task and cocaine task for brain activity	Comparative, neuroimaging study	N = 91 (n=59 CU, n=32 H)	Cocaine	Images	Nonclinical	7	Visual
**Moeller et al. 2013**	Gene testing	Comparative, neuroimaging study	N=120 (n=73 CD, n=47 H)	Cocaine	Images and words	Clinical and nonclinical	7	Visual
**Mongeau-Pérusse et al. 2021**	Assess cue-induced craving	Single-site double-blind randomised controlled superiority trial	N = 78	Cocaine	Script	Clinical and nonclinical	7.5	Auditory
**Montgomery et al. 2010**	Cocaine task for attentional bias and assess cue-induced craving	Double-blind, placebo-controlled trial	N = 72 (n=32 CU, n=40 HC)	Cocaine	Drug attentional bias task (Stroop test) and images	Nonclinical	7	Visual
**Moran-Santa et al. 2014**	To assess medication as part of cocaine treatment	Double-blind, placebo-controlled cross-over study	N = 62	Cocaine	Paraphernalia, script and videos	Nonclinical	7	Visual Tactile
**Moran-Santa et al. 2015**	To assess medication as part of cocaine treatment	Double-blind, placebo-controlled trial	N = 84	Cocaine	Paraphernalia	Nonclinical	7.5	Visual Tactile
**Moran-Santa et al. 2018**	To assess medication as part of cocaine treatment	Double-blind, placebo-controlled cross-over study	N = 25	Cocaine	Paraphernalia and recall a memory of cocaine use	Nonclinical	7.5	Visual Auditory Tactile
**Moscon et al. 2016**	Cocaine task for brain activity	Exploratory open trial	N = 18 (n=9 crack users, n=9 HC)	Crack	Paraphernalia and videos	Nonclinical	6.5	Visual

**Negrete & Emil 1992**	Assess cue-induced craving	Comparative, exploratory study	N = 52 (n=36 CU, n=16 age and gender matched controls)	Cocaine	Images	Clinical	6.5	Visual
**Parvaz et al. 2016**	Assess cue-induced craving	Laboratory-based, cross-sectional study	N = 76	Cocaine	Images	Nonclinical	7	Visual
**Parvaz et al. 2017**	Cocaine task for brain activity and cocaine task for attentional bias	Comparative neuroimaging study	N = 19	Cocaine	Images	Nonclinical	7	Visual
**Parvaz et al. 2021**	Cocaine task for brain activity	Comparative neuroimaging study	N = 58 (n=30 CU, n=28 H)	Cocaine	Images	Nonclinical	7	Visual
**Penetar et al. 2012**	Assessing treatment for cocaine dependence (with the use of cues)	Within-subject, single-blind study	N = 20 (n=9 CU, n=11 cannabis dependent)	Cocaine	Images and paraphernalia	Nonclinical	6.5	Visual Tactile
**Petrakis et al. 1996**	To assess medication as part of cocaine treatment	Randomised, double-blind, cross-over study	N = 20	Cocaine	Paraphernalia and videos	Clinical	6.5	Visual Tactile
**Pettorruso et al. 2019**	Assessing treatment for cocaine dependence (with the use of cues)	Double-blind, sham-controlled, neuro-navigated rTMS study design	N = 20	Cocaine	Videos	Nonclinical	6.5	Visual
**Pike et al. 2013**	Assess inhibitory control	Between-subjects study	N = 30	Cocaine	Images	Nonclinical	6.5	Visual
**Potenza et al. 2012**	Cocaine task for brain activity	Exploratory neuroimaging study	N = 66 (n=30 CU, n=36 alcohol)	Cocaine	Scripts	Nonclinical	6.5	Auditory
**Price et al. 2009**	To assess medication as part of cocaine treatment	Randomised, double-blind, placebo-controlled trial	N = 10	Cocaine	Paraphernalia	Nonclinical	7.5	Tactile Visual

**Price et al. 2013**	To assess medication as part of cocaine treatment	Randomised, placebo-controlled laboratory study	N = 32	Cocaine	Paraphernalia, script and videos	Clinical and nonclinical	7.5	Visual Auditory Tactile
**Prisciandaro et al. 2013**	Cocaine task for brain activity	Random placebo-controlled trial	N = 30	Cocaine	Images and paraphernalia	Nonclinical	7.5	Visual Tactile
**Prisciandaro et al. 2013**	To assess medication as part of cocaine treatment	Random placebo-controlled study	N = 25	Cocaine	Images	Nonclinical	8	Visual
**Prisciandaro et al. 2014**	Cocaine task for brain activity	Exploratory neuroimaging study	N = 38	Cocaine	Images	Clinical and nonclinical	7	Visual
**Prisciandaro et al. 2014**	Cocaine task for brain activity	Cross-sectional study	N = 41	Cocaine	Images and paraphernalia	Nonclinical	6.5	Visual Tactile
**Ray et al. 2014**	To assess memory recall	Exploratory study	N = 35 (n=14 inpatients, n=21 young adults with limited experience)	Cocaine	Images	Clinical and nonclinical	6	Visual
**Ray et al. 2015**	Cocaine task for brain activity	Comparative, neuroimaging study	N = 28 (n=15 crack, n=13 H)	Crack	Images	Nonclinical	6.5	Visual
**Regier et al. 2021**	Cocaine task for brain activity	Neuroimaging study	N = 73	Cocaine	Images	Clinical	6.5	Visual
**Reid et al. 1998**	Assess cue-induced craving	Double-blind, randomised, counterbalanced study	N = 22	Cocaine (recent crack use)	Drug preparation task, paraphernalia, smell and videos	Clinical	7.5	Visual Auditory Tactile Olfactory
**Reid et al. 1999**	To assess medication as part of cocaine treatment	Double-blind, randomised,	N = 23	Crack	Drug preparation task,	Clinical	7	Visual Auditory Tactile

		counterbalanced design			paraphernalia, smell, and videos			Olfactory
**Reid et al. 2003**	Cocaine task for brain activity	Exploratory, neuroimaging study	N= 24	Crack	Paraphernalia, script and videos	Clinical	6.5	Visual Auditory Tactile
**Reid et al. 2004**	To assess cocaine induced psychosis	Exploratory study	N = 23	Cocaine	Paraphernalia, script, smell and videos	Clinical	6.5	Visual Auditory Tactile Olfactory
**Reid et al. 2006**	Cocaine task for brain activity	Within-subject, cross-over study	N = 13	Crack	Drug use and paraphernalia	Clinical	6	Visual Tactile Gustatory
**Reid et al. 2008**	Cocaine task for brain activity	Within-subject, cross-over study	N = 13	Crack	Paraphernalia, script and videos	Nonclinical	6.5	Visual Auditory Tactile
**Reid & Thakkar 2009**	To assess medication as part of cocaine treatment	Randomised, placebo-controlled, within-subjects, crossover study	N = 20	Crack	Paraphernalia, smell, videos	Nonclinical	6.5	Visual Auditory Tactile Olfactory
**Renshaw et al. 1999**	Cocaine task for brain activity	Randomised, double-blind, placebo-controlled study	N = 14	Cocaine	Videos	Nonclinical	7.5	Visual
**Robbins et al. 1992**	To assess medication as part of cocaine treatment	Randomised, double-blind, placebo-	N = 20	Cocaine	Audiotape, drug preparation task and videos	Clinical	7.5	Visual Auditory Tactile

		controlled study						
**Robbins et al., 1997**	Assess cue-induced craving	Exploratory study	N = 150	Cocaine	Audiotape, paraphernalia and videos	Clinical	7	Visual Auditory Tactile
**Robbins et al. 1999**	Assess cue-induced craving	Exploratory study	N = 38	Cocaine	Audiotape, drug preparation task and videos	Clinical	6.5	Visual Auditory Tactile
**Robbins et al. 2000**	Assess cue-induced craving	Exploratory study	N = 81	Cocaine	Audiotape, drug preparation task, paraphernalia and videos	Clinical	6.5	Visual Auditory Tactile
**Rohsenow et al. 2004**	Assessing treatment for cocaine dependence (with the use of cues)	Experimental 2 x 2 study	N = 165	Cocaine	Audiotape	Clinical	8	Auditory
**Rohsenow et al. 2007**	Assess cue impact on drug use	Exploratory study	N = 163	Cocaine	Audiotapes	Clinical	7	Auditory
**Rosse et al. 1994**	To assess medication as part of cocaine treatment	Double-blind, placebo-controlled study	N = 66	Cocaine	Images	Clinical	7.5	Visual
**Rosse et al. 1995**	Cocaine task for eye-tracking	Exploratory study	N = 44	Crack	Images	Clinical	6.5	Visual
**Rosse et al. 1997**	Cocaine task for eye-tracking	Exploratory study	N = 19	Crack	Images	Clinical	6	Visual
**Roy et al. 2002**	Assess the relationship between central monoamine metabolites and craving cocaine	Exploratory study	N = 20	Cocaine	Videos	Clinical	6.5	Visual
**Saladin et al. 2006**	To test cocaine cues in virtual reality	Feasibility, exploratory study	N = 11	Crack	VR drug environments	Nonclinical	6.5	Visual Auditory
**Saladin et al. 2013**	To assess medication as part of cocaine treatment	Randomised, double-blind, placebo-controlled study	N = 50	Cocaine	Paraphernalia and videos	Nonclinical	7.5	Visual Tactile

**Santa Ana et al. 2015**	To assess medication as part of cocaine treatment	Randomised, double-blind, placebo-controlled pilot study	N = 47	Cocaine	Paraphernalia	Clinical and nonclinical	8	Visual Tactile
**Satel et al. 1995**	To assess medication as part of cocaine treatment and assess cue-induced craving	Counterbalanced, double-blind study	N = 25	Cocaine	Paraphernalia and videos	Clinical	7	Visual Tactile
**Scala et al. 2024**	Cocaine task for brain activity	Cross-sectional, neuroimaging study	N = 41 (n=21 ND CU, n=20 controls)	Cocaine	Videos	Nonclinical	6.5	Visual
**Schlauch et al. 2013**	Assess cue-induced craving	Experimental study	N = 155	Cocaine	Images	Clinical (criminal)	7	Visual
**Schulte et al. 2019**	To assess medication as part of cocaine treatment	Randomised, double-blind study	N = 24	Cocaine snorting only	Images	Nonclinical	7	Visual
**Sinha et al. 1999**	Assess cue-induced craving	Exploratory study	N = 10 (Study 1), N = 10 (Study 2)	Cocaine	Script	Clinical	6.5	Auditory
**Sinha et al. 2000**	Assess cue-induced craving	Exploratory study	N = 20	Cocaine	Script	Clinical	6.5	Auditory
**Sinha et al. 2003**	Assess cue-induced craving	Exploratory study	N = 54	Cocaine	Script	Clinical	6.5	Auditory
**Sinha et al. 2005**	Cocaine task for brain activity	Comparative neuroimaging study	N = 20	Cocaine	Script	Clinical	6.5	Auditory
**Sinha et al. 2006**	Assess cue-induced craving	Prospective study	N = 49	Cocaine	Script	Clinical	7	Auditory
**Sinha et al. 2007**	Assess cue-induced craving	Exploratory study	N = 19	Cocaine	Script	Nonclinical	6.5	Auditory
**Smelson et al. 1997**	To assess medication as part of cocaine treatment	Exploratory study	N = 4	Cocaine	Videos	Clinical	6.5	Visual
**Smelson et al. 1998**	Assess cue-induced craving	Exploratory study	N = 14	Cocaine	Videos	Clinical	6.5	Visual

**Smelson et al. 2002**	To assess medication as part of cocaine treatment	6-week, open-label pilot study	N = 18	Cocaine	Videos	Clinical	6.5	Visual
**Smelson et al. 2002**	Assess cue-induced craving	Exploratory study	N = 91	Cocaine	Videos	Clinical	6.5	Visual
**Smelson et al. 2004**	To assess medication as part of cocaine treatment	Randomised, double-blind, placebo-controlled pilot study	N = 34	Cocaine	Paraphernalia and videos	Clinical	7.5	Visual Tactile
**Smelson et al. 2006**	To assess medication as part of cocaine treatment	Randomised, double-blind, placebo-controlled trial	N = 31	Cocaine	Videos	Clinical	7.5	Visual
**Smelson et al. 2012**	Assess cue-induced craving and for gene testing	Exploratory study	N = 34	Cocaine	Paraphernalia and videos	Clinical	6.5	Visual Tactile
**Smelson et al. 2013**	Assessing treatment for cocaine dependence (with the use of cues)	Randomised, double-blind, placebo-controlled trial	N = 101	Cocaine	Paraphernalia and videos	Clinical	7.5	Visual Tactile
**Smith et al. 2014**	Cocaine task for brain activity	Comparative, neuroimaging study	N = 129 (n=27 recreational, n=50 stimulant dependent, n=52 H)	Cocaine	Drug attentional bias task (Stroop test)	Nonclinical	6.5	Visual
**Smith et al. 2020**	Cocaine task for attentional bias	Comparative, exploratory study	N = 92 (n=40 CD, n=24 abstinent former CD, n=28 HC)	Cocaine	Drug attentional bias task (Stroop test)	Clinical	6.5	Visual
**Smith et al. 2023**	Cocaine task for brain activity	Exploratory, neuroimaging study	N = 72	Cocaine	Script	Clinical	6.5	Auditory
**Sokhadze et al. 2008**	Cocaine task for brain activity	Comparative, neuroimaging study	N = 14	Cocaine	Images	Nonclinical	6.5	Visual

**Stauffer et al. 2016**	To assess medication as part of cocaine treatment	Experimental, pilot study	N = 22	Opioid	Videos	Clinical	7.5	Visual
**Sterling et al. 2004**	Assess cue-induced craving	Experimental, 2 x 4 repeated measures study	N = 81	Cocaine	Videos	Nonclinical	7.5	Visual
**Strickland et al. 2016**	Assessing treatment for cocaine dependence (with the use of cues)	Exploratory study	N = 44	Cocaine	Images	Nonclinical	7	Visual
**Strickland et al. 2018**	To assess choice in a monetary task	Exploratory study	N = 12	Cocaine	Images	Nonclinical	6.5	Visual
**Tap et al. 2024**	Cocaine task for brain activity	Comparative, neuroimaging study	N = 111 (n=57 CU, n=54 controls)	Cocaine snorting only	Images	Nonclinical	6.5	Visual
**Tull et al. 2011**	Cocaine task for attentional bias	Exploratory 2 x 2 x 2 study	N = 60 (n=30 PTSD, n=30 without PTSD)	Cocaine	Images and script	Clinical	6.5	Visual Auditory
**Tull et al. 2016**	Assess cue-induced craving	Exploratory study	N = 42	Cocaine	Images	Clinical	6.5	Visual
**Vaccaro et al. 2024**	Cocaine task for brain activity	Comparative, neuroimaging study	N = 103 (n=30 CD, n=28 PG, n=45 control)	Cocaine	Videos	Nonclinical	6.5	Visual
**Vadhan et al. 2007**	Cocaine task for attentional bias	Comparative, exploratory study	N = 37 (n=17 treatment seeking, n=20 non treatment seeking)	Cocaine	Drug attentional bias task (Stroop test)	Clinical and nonclinical	6	Visual
**Van de Laar et al. 2004**	Cocaine task for brain activity	Comparative, exploratory study	N = 46 (n=26 CU, n=20 H)	Cocaine	Images	Clinical	7	Visual
**Verveer et al. 2020**	Assessing treatment for cocaine dependence (with the use of cues)	Randomised, sham-controlled tDCS study	N = 59	Cocaine	Images	Clinical	7.5	Visual
**Vincent et al. 2018**	Cocaine task for brain activity	Exploratory, neuroimaging study	N = 54	Cocaine	Images	Nonclinical (criminal)	6	Visual

**Volkow et al. 2006**	Cocaine task for brain activity	Exploratory, neuroimaging study	N = 18	Cocaine	Videos	Nonclinical	6.5	Visual
**Volkow et al. 2008**	Cocaine task for brain activity	Exploratory, neuroimaging study	N = 20	Cocaine	Images	Nonclinical	6.5	Visual
**Volkow et al. 2010**	Cocaine task for brain activity	Exploratory, neuroimaging study	N = 24	Cocaine	Videos	Nonclinical	6.5	Visual
**Volkow et al. 2010**	Cocaine task for brain activity	Placebo-controlled, cross over, neuroimaging study	N = 24	Cocaine	Videos	Nonclinical	6.5	Visual Auditory
**Volkow et al. 2011**	Cocaine task for brain activity	Exploratory, neuroimaging study	N = 26	Cocaine	Videos	Nonclinical	7	Visual
**Volkow et al. 2014**	Cocaine task for brain activity	Comparative, neuroimaging study	N = 62 (n=43 CU, n=19 H)	Cocaine	Videos	Nonclinical	6.5	Visual
**Waldrop et al. 2010**	Assess cue-induced craving	Exploratory study	N = 100 (n=47 controls, n=53 CD)	Cocaine	Paraphernalia and videos	Nonclinical	7	Visual Auditory Tactile
**Wang et al. 2021**	Cocaine task for brain activity	Comparative, neuroimaging study	N = 103 (n=44 CU, n=59 nonusers)	Cocaine	Images	Nonclinical	7	Visual
**Waters et al. 2012**	Assess cue-induced craving	Exploratory study	N = 68	Opioid	Drug attentional bias task (Stroop test)	Clinical	7	Visual
**Webber et al. 2021**	Cocaine task for brain activity	Exploratory, neuroimaging study	N = 52	Cocaine	Images	Nonclinical	6.5	Visual
**Webber et al. 2021**	Cocaine task for brain activity and cocaine task for eye-tracking	Exploratory, neuroimaging study	N = 43	Cocaine	Images	Nonclinical	6.5	Visual

**Wexler et al. 2001**	Cocaine task for brain activity	Comparative, neuroimaging study	N = 32 (n=11 CU, n=21 controls)	Cocaine	Videos	Nonclinical	6.5	Visual Auditory
**Wilcox et al. 2011**	Cocaine task for brain activity	Comparative, neuroimaging study	N = 28 (n=14 CU, n=14 = matched controls)	Cocaine	Videos	Nonclinical	7	Visual
**Wong et al. 2006**	Cocaine task for brain activity	Exploratory, neuroimaging study	N = 19	Cocaine	Audiotape and videos	Nonclinical	7	Visual Auditory
**Xu et al. 2013**	Cocaine task for brain activity	Exploratory, neuroimaging study	N = 67	Cocaine	Script	Clinical	6.5	Auditory
**Young et al. 2014**	Cocaine task for brain activity	Exploratory, neuroimaging study	N = 23	Cocaine (smoking)	Images	Nonclinical	8	Visual
**Zhang et al. 2019**	Cocaine task for brain activity	Comparative, neuroimaging study	N = 44 (n=20 CD, n=24 H BMI matched controls)	Cocaine	Images	Nonclinical	7	Visual
**Zhang et al. 2020**	Cocaine task for brain activity	Exploratory, neuroimaging study	N = 52	Cocaine	Images	Nonclinical	7	Visual
**Zhang et al. 2020**	Cocaine task for brain activity	Exploratory, neuroimaging study	N = 23	Cocaine	Images	Nonclinical	7	Visual

*Jinks et al., 2019 assessment scale for quality appraisal

AD = Alcohol Dependent
BPD = Borderline Personality Disorder
CD = Cocaine Dependent
CU = Cocaine User
GD = Gambling Dependent
H = Healthy
HC = Healthy Control

ND = Non-Dependent
NW = No withdrawal
PG = Problem Gambler
W = Withdrawal
